# Monitoring COPD patients: systemic and bronchial eosinophilic inflammation in a 2-year follow-up

**DOI:** 10.1186/s12890-024-03062-1

**Published:** 2024-05-19

**Authors:** Patrizia Pignatti, Dina Visca, Martina Zappa, Elisabetta Zampogna, Laura Saderi, Giovanni Sotgiu, Rosella Centis, Giovanni Battista Migliori, Antonio Spanevello

**Affiliations:** 1 Allergy and Immunology Unit, Istituti Clinici Scientifici Maugeri, IRCCS, Via S.Maugeri 10, Pavia, 27100 Italy; 2 Division of Pulmonary Rehabilitation, Istituti Clinici Scientifici Maugeri, IRCCS, Tradate, Italy; 3 Department of Medicine and Surgery, Respiratory Diseases, University of Insubria, Varese- Como, Italy; 4 Clinical Epidemiology and Medical Statistics Unit, Department of Biomedical Medicine, Surgery and Pharmacy Sciences, University of Sassari, Sassari, Italy; 5 Respiratory Diseases Clinical Epidemiology Unit, Istituti Clinici Scientifici Maugeri, IRCCS, Tradate, Italy

**Keywords:** Sputum, Eosinophils, Blood, Inhaled corticosteroids, COPD

## Abstract

**Background:**

High blood eosinophils seem to predict exacerbations and response to inhaled corticosteroids (ICS) treatment in patients with chronic obstructive pulmonary disease (COPD). The aim of our study was to prospectively evaluate for 2 years, blood and sputum eosinophils in COPD patients treated with bronchodilators only at recruitment.

**Methods:**

COPD patients in stable condition treated with bronchodilators only underwent monitoring of lung function, blood and sputum eosinophils, exacerbations and comorbidities every 6 months for 2 years. ICS was added during follow-up when symptoms worsened.

**Results:**

63 COPD patients were enrolled: 53 were followed for 1 year, 41 for 2 years, 10 dropped-out. After 2 years, ICS was added in 12/41 patients (29%) without any statistically significant difference at time points considered. Blood and sputum eosinophils did not change during follow-up. Only FEV_1_/FVC at T0 was predictive of ICS addition during the 2 year-follow-up (OR:0.91; 95% CI: 0.83–0.99, *p* = 0.03). ICS addition did not impact on delta (T24-T0) FEV_1_, blood and sputum eosinophils and exacerbations. After 2 years, patients who received ICS had higher blood eosinophils than those in bronchodilator therapy (*p* = 0.042). Patients with history of ischemic heart disease increased blood eosinophils after 2 years [*p* = 0.03 for both percentage and counts].

**Conclusions:**

Blood and sputum eosinophils remained stable during the 2 year follow-up and were not associated with worsened symptoms or exacerbations. Almost 30% of mild/moderate COPD patients in bronchodilator therapy at enrollment, received ICS for worsened symptoms in a 2 year-follow-up and only FEV_1_/FVC at T0 seems to predict this addition. History of ischemic heart disease seems to be associated with a progressive increase of blood eosinophils.

## Introduction

Chronic obstructive pulmonary disease (COPD) is primarily caused by smoking habits, even if it can also occur in non-smoking subjects. COPD is characterized by neutrophilic airway inflammation; however, from 20 to 30% of patients present increased eosinophils in central and small airways [1]. Blood eosinophils, usually, reflect airway eosinophils, although mildly correlate with sputum eosinophils, and when increased, represent a treatable trait in COPD patients [2]. They are a biomarker related to the risk of exacerbations and a predictor of efficacy of inhaled corticosteroid treatment (ICS) [3–6]. Low blood eosinophils (< 100/mcL) identify patients with a higher risk of bacterial exacerbations and with ineffective treatment with ICS, while patients with blood eosinophils ≥ 300 /mcL should be those with the best response to this treatment [1]. Findings on the role of blood eosinophils as predictor of future exacerbations are discordant, depending on the different characteristics of the subjects enrolled in the studies and on their previous use of ICS. Tan et al. reported that high blood eosinophils are associated with a rapid forced expiratory volume in the first second (FEV_1_) decline both in COPD and in healthy controls [7]. The meaning of increased sputum eosinophils is less clear in COPD patients than in those with asthma. Some studies showed that patients with increased sputum eosinophils have higher air trapping and emphysema others showed that airway eosinophils are not related to lung deterioration [8, 9].

Prospective studies periodically evaluating and following-up sputum other than blood eosinophils for a relative long period in mild-moderate COPD patients are presently scarce.

The aim of the present study was to evaluate a cohort of mild-moderate COPD patients, not treated with ICS at baseline, in a follow-up period of one and/or two years with regards to variation of blood and sputum eosinophils during follow-up.

## Materials and methods

### Study participants

We prospectically enrolled consecutive COPD patients referred to the Division of Pulmonary Rehabilitation of the Istituti Clinici Scientifici Maugeri (Tradate, Italy) from 2017 to 2020, in stable condition and on regular treatment with single long-acting β2-agonist (LABA) or long-acting muscarinic antagonists (LAMA) or dual bronchodilators (LABA + LAMA), without ICS treatment for at least one month. COPD was diagnosed according to Global Initiative for Chronic Obstructive Lung Disease (GOLD) criteria [1]. Patients with a previous asthma diagnosis were excluded. None of the patients had infections of the upper respiratory tract or exacerbations in the previous 2 months. A written informed consent was signed by each enrolled patient. This study conformed to the declaration of Helsinki and was approved by the Internal Review Board of Istituti Clinici Scientifici Maugeri (number 2126/2017 CE) and registered on ClinicalTrials.gov. NCT05795712.

### Study design

Medical history and comorbidities were recorded at T0 (baseline) and blood and sputum eosinophils evaluated. Patients were monitored every 6 months for one or two years (T6, T12, T18 and T24). Blood and sputum eosinophils were re-evaluated at each time point as well as lung function, history of exacerbations and health status assessed with COPD Assessment Test (CAT). During follow-up, ICS was added by a respiratory specialist, according to increase of symptoms and exacerbations, without considering blood or sputum eosinophil amount as reported in Global Strategy for the Diagnosis, Management and Prevention of COPD, Global Initiative for Chronic Obstructive Lung Disease (GOLD) 2017 [10].

### Blood cell evaluation and sputum induction

Peripheral blood eosinophils were determined using UniCelDxH 800 haematology analyser (Beckman Coulter, Pasadena, CA) for cell differentiation.

Sputum was induced and processed according to ERS Statement and to previous studies [2, 11]. Sputum eosinophilia was defined when sputum eosinophils were ≥ 3% [12].

### Lung function

Lung function was evaluated through spirometry (Pony FX Spirometer, Cosmed, Chicago, IL, USA) according to standards [13]. FEV_1_, forced vital capacity (FVC), and FEV_1_/FVC were recorded. Positive bronchial reversibility test was defined as an increase of at least 12% in FEV_1_ after inhalation of 400mcg of bronchodilator and improvement in absolute value of more than 200mL [14].

### Statistical analysis

Qualitative and quantitative variables were collected in an anonymized electronic database and expressed as media ± standard deviation or median interquartile range according to their distribution. The chi-square or Fisher’s exact test were used to compare qualitative variables between two independent groups, whereas the Student’s t and the Mann-Whitney tests were used for comparing quantitative variables, following their parametric or non-parametric distribution, respectively. For comparing quantitative variables across different time points, ANOVA or the Friedman test was used. Logistic regression analysis was performed to identify predictive factors for the addition of ICS. Spearman’s correlation was used for correlations between data. A p-value < 0.05 was considered statistically significant. The statistical software used was STATA 17.

## Results

We recruited 63 COPD patients, 53 were followed for 1 year (96.6% with suitable sputum samples), 41 for 2 years (82.9% with suitable sputum samples). Drop-out before the first year (10/63, 15.9%) was mainly due to cardiovascular diseases; these patients had significantly higher blood eosinophil count at baseline than those who were followed for 2 years (241.2 eos/mcL, IQR = 182.7-348.3 and 153.7 eos/mcL, IQR = 94.6-229.4, respectively; *p* = 0.04). We only analyzed data of patients who reached at least 1 year-follow-up.

Patients’ characteristics are shown in Table 1.

**Table 1:** Patients’ characteristics at baseline (T0)

T0			*n* = 53
Age, years			72.7 (7.0)
BMI, kg/m^2^			28 (5.1)
Smoking status, n (%)	Non-smoker		3 (5.7)
	Current smoker		20 (37.7)
	Former smoker		30 (56.6)
Therapy, n (%)	LAMA		19 (35.9)
	LABA		2 (3.8)
	LAMA-LABA		32 (60.4)
CAT			8.2 (5.6)
Step GOLD		1 2 3	17 (32.1) 30 (56.6) 6 (11.3)
Grade		A B C D	28 (52.8) 18 (34.0) 5 (9.4) 2 (3.8)
Blood leucocytes, mmc			6.7 (1.5)
Blood neutrophils, %			56.5 (9.2)
Blood lymphocytes, %			30.1 (8.1)
Blood eosinophils, %			2.9 (1.5)
Blood eosinophils/mcL			182.7 (104.4-241.2)
Exacerbations previous year, n			0 (0–1)
FEV_1_, L			1.9 (0.6)
FEV_1_, %			71.3 (19.3)
FVC; L			3.2 (0.9)
FVC, %			95 (81–110)
FEV_1_/FVC			59 (53–62)
Sputum total cells, x10^4^/ml			228 (79–346)
Viability, %			90.3 (77.6–93.4)
Sputum macrophages, %			15.5 (8.8–24.2)
Sputum neutrophils, %			75.8 (60–84)
Sputum eosinophils, %			1.7 (0.7–3.4)
Sputum lymphocytes, %			1.1 (0.6–1.8)
Sputum bronchial epithelial cells, %			3.2 (1.2–5.1)
**Comorbidities**			
Ischemic heart disease, n (%)			11 (20.8)
Atrial fibrillation, n (%)			9 (17.0)
Hypertension, n (%)			29 (54.7)
OSAS, n (%)			16 (30.2)
Obesity, n (%)			12 (22.6)

Legend: Data are expressed as mean (Standard Deviation: SD) or median (interquartile range: IQR) according to their distribution. BMI = body mass index; CAT = COPD (Chronic Obstructive Pulmonary Disease) assessment test; FEV_1_ = forced expiratory volume in the first second; L = Liters; FVC = forced vital capacity; OSAS = obstructive sleep apnea syndrome.

Patients, according to inclusion criteria, were mainly GOLD step 1 and 2 (88.7%) and grade A or B (86.8%). The small percentage of subjects in stage 3 (11.3%) did not differ from the other patients as concerns airway or systemic inflammation. Even if we excluded from recruitment patients with history of asthma, 18.9% of the subjects had a significant bronchodilator response at baseline, with FEV_1_/FVC still ≤ 70%, without any differences in the other baseline variables compared with patients with negative bronchodilator response. Considering the algorithm of GOLD document for the individualized assessment of symptoms and exacerbation risk [1], at the enrolment none of the patients was in group E (≥ 2 moderate exacerbations or ≥ 1 severe and blood eosinophils ≥ 300) therefore candidate for the addition of ICS, according to recent GOLD document [1]. 9/53 patients received ICS treatment in the past before the enrollment in the study, no differences were found compared with ICS-naïve patients in history of exacerbations, blood or sputum eosinophils at T0, or at the different time points of the study.

### Treatment follow-up

**Fig. 1:**
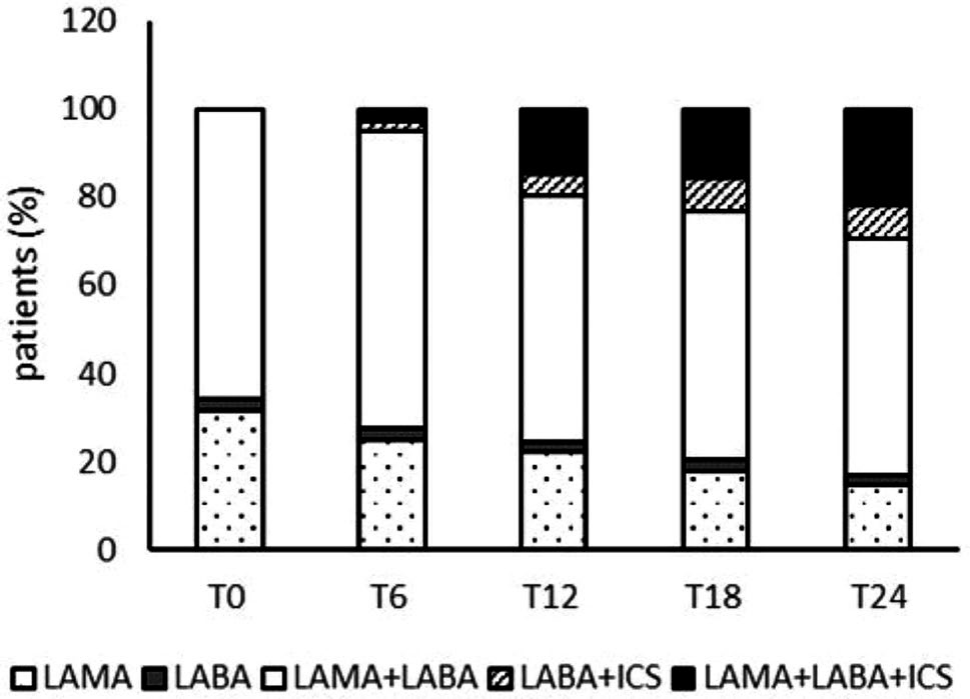
Treatment received by patients who were followed every six months for 2 years. ICS was added according to increased symptoms *Legend*: LAMA = long-acting muscarinic antagonists; LABA = long-acting beta2-agonist; ICS = inhaled corticosteroids

After 1 year, ICS (as ICS + LABA or triple therapy) was added for increased respiratory symptoms in 10/53 subjects (18.9%) and after 2 years in 12/41 patients (29.3%) with a clear trend but without any statistically significant difference among the time points considered: T6, T12, T18 and T24, Fig. 1. Considering all the variables at T0, only FEV_1_% was significant in a univariate model for the prediction of ICS addition after one year, and FEV_1_/FVC for the addition after two years, Table 2.

Delta (T24-T0) of: FEV_1_, blood and sputum eosinophils and exacerbations were not different between patients with or without addition of ICS.

**Table 2:** Baseline variables predictive of ICS addition during the 2 year-follow-up (12/41 subjects, 29.3%, received ICS)

Baseline variables		OR (95% CI)	*p*-value
Age, years		0.97 (0.88–1.07)	0.54
BMI, kg/m2		0.96 (0.84–1.10)	0.57
Smoking status	Non-smoker	-	-
	Current smoker	0.13 (0.01–1.89)	0.13
	Former smoker	0.22 (0.02–2.83)	0.24
CAT		1.07 (0.94–1.20)	0.30
Number of exacerbations previous year		1.19 (0.52–2.72)	0.68
FEV_1_, L		0.49 (0.13–1.80)	0.28
FEV_1_, %		0.97 (0.93–1.01)	0.14
FVC; L		1.05 (0.48–2.29)	0.90
FVC, %		0.99 (0.96–1.03)	0.71
FEV_1_/FVC		0.91 (0.83–0.99)	**0.03**
Blood leucocytes, %		1.04 (0.67–1.62)	0.86
Blood neutrophils, %		1.00 (0.92–1.08)	0.91
Blood lymphocytes, %		1.02 (0.94–1.11)	0.68
Blood eosinophils, %		1.06 (0.67–1.67)	0.80
Blood eosinophils/mcL		1.00 (1.00-1.01)	0.76
Sputum neutrophils, %		1.01 (0.97–1.05)	0.70
Sputum eosinophils, %		1.08 (0.94–1.23)	0.27
**Comorbidities**			
Ischemic heart disease		1.60 (0.32–8.11)	0.57
Atrial fibrillation		0.57 (0.06–5.69)	0.63
Hypertension		0.53 (0.13–2.06)	0.36
OSAS		0.15 (0.02–1.32)	0.09
Obesity		0.53 (0.09–2.94)	0.46

Legend: ICS = inhaled corticosteroids; OR = odds ratio; CI = Confidence Intervals; BMI = body mass index; CAT = COPD assessment test; FEV_1_ = forced expiratory volume in the first second; L = Liters; FVC = forced vital capacity; OSAS = obstructive sleep apnea syndrome

### Lung function

FEV_1_/FVC was significantly different at T0, T12, and T24 (*p* = 0.01). Post hoc analysis found a statistically significant difference between T0 vs. T12 (*p* = 0.04) and T0 vs. T24 (*P* = 0.012), median FEV_1_/FVC T0 = 59.0, IQR: 51.0–62.0; T12: 60.0, IQR: 53–64.0; T24: 60.0, IQR: 51.1–65.8. Delta (T24-T0) FEV_1_ was comparable in current (37.7%) and former (56.6%) smokers. Delta FEV_1_ L (T24-T0) in ICS treated patients was 0.055 L (IQR: -0.55- -0.55) and − 0.65 L (IQR: -1.56—1.56) in ICS not treated patients, although the difference was not statistically significant.

### Blood and sputum eosinophils

**Fig. 2:**
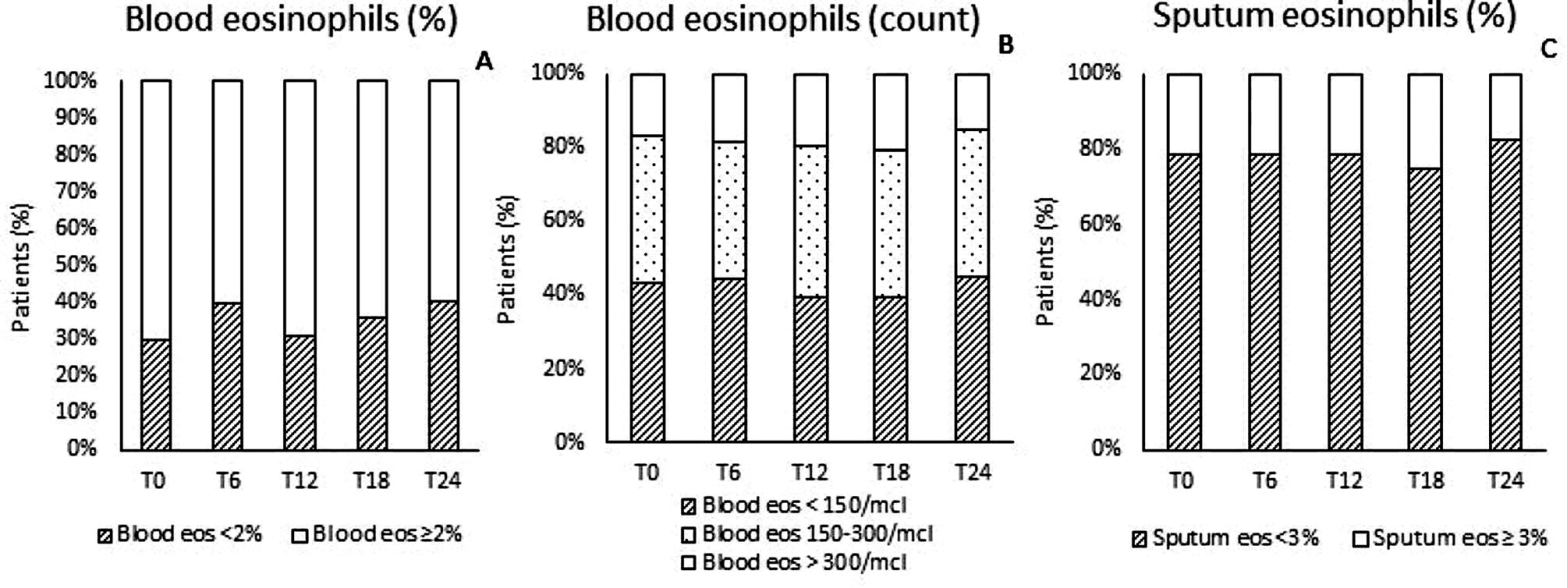
Percentage of subjects monitored for 2 years with blood eosinophils < or ≥ 2%(panel **A**), < 150 eos/mcL or 150–300 eos/mcL or > 300 eos/mcL (panel **B**) and with sputum eosinophils < or ≥ 3% (panel **C**)

At T0, 70.0% of subjects had blood eosinophils ≥ 2%, 56.6% ≥150 eos/mcL and 17.0% ≥300 eos/mcL, 28.3% had sputum eosinophils ≥ 3, Fig. 2 shows subjects, followed for 2 years. At baseline sputum eosinophils and CAT values mildly correlated (*r* = 0.29, *p* = 0.04). No significant differences in blood (both as % and as count) and sputum eosinophils were found at the different time points, Table 3.

**Table 3:** Blood and sputum eosinophils in patients followed for 2 years

	T0	T6	T12	T18	T24
Blood eosinophils %	2.9 (1.5)	2.8 (1.9)	2.7 (1.6)	2.8 (1.7)	2.7 (1.8)
Blood eosinophils/mcL	153.6 (106.9-233.4)	150.6 (91.7-326.4)	174.3 (115.2-202.5)	178.1 (104.3-213.6)	149.3 (91.3-214.4)
Sputum eosinophils %	1.8 (0.7–4.4)	1.2 (0.4–3.7)	1.0 (0.6–3.7)	1.0 (0.6–2.6)	1.3 (0.7–2.8)

Legend: Data are expressed as mean (Standard Deviation: SD) or median (interquartile range: IQR) according to their distribution

Frequency of patients with sputum eosinophils ≥ 3% was comparable at the different time points (T0 = 28.3%, T6 = 37.3%, T12 = 34.7%, T18 = 24.8% T24 = 20.6%, *p* = 0.52), in Fig. 2 are represented frequencies of subjects followed for 2 years. Blood and sputum eosinophils were mildly correlated at T0 (rho = 0.414, *p* = 0.001 blood eosinophils % vs. sputum eosinophils and rho 0.439, *p* = 0.001, blood eosinophil counts vs. sputum eosinophils).

At T24, patients with blood eosinophils ≥ 2% or ≥ 150 eos/mcL did not differ from those with lower eosinophils as concerns delta FEV_1_, delta CAT and total exacerbations. When we divided patients according to blood eosinophils < 100/mcL and in the range 100–300/mcL no differences were found in the variables reported in Table 4.

**Table 4:** Analysis of patients divided according to their amount of blood eosinophils at T0

	Blood eos T0		*p*-value
	< 100	100–300	
Exacerbations at T24	0 (0–1)	0 (0–1)	0.88
Sputum eosinophils at T24	0.8 (0.6–2.3)	1.4 (0.7–2.8)	0.52
Delta (T24-T0) blood eos	24 (-2; 27)	-7.5 (-29; 43.5)	0.56
Delta (T24-T0) sputum eos	0 (-1; 1)	0.5 (-2; 1)	0.78
Delta (T24-T0) FEV_1_ L	-0.03 (0.2)	-0.06 (0.2)	0.66

Legend: Data are expressed as mean (Standard Deviation: SD) or median (interquartile range: IQR) according to their distribution

At T24 patients who received ICS for increased symptoms had higher blood eosinophils than those who only maintained bronchodilator therapy (median 233 eos/mcL IQR 113–338 and median 144 IQR 86–144, respectively, *p* = 0.042) with comparable blood eosinophils at T0. A tendency towards a reduction of sputum eosinophils (delta T24-T0) in ICS treated subjects after 2 years was present without any statistically significant difference ( -1 (-4.4; 0.4) in ICS treated vs. 0.7 (-0.9; 1.4) in ICS not treated subjects, *p* = 0.12). Delta (T24-T0) blood and sputum eosinophils were comparable in current and former smokers.

### Exacerbations

**Fig. 3:**
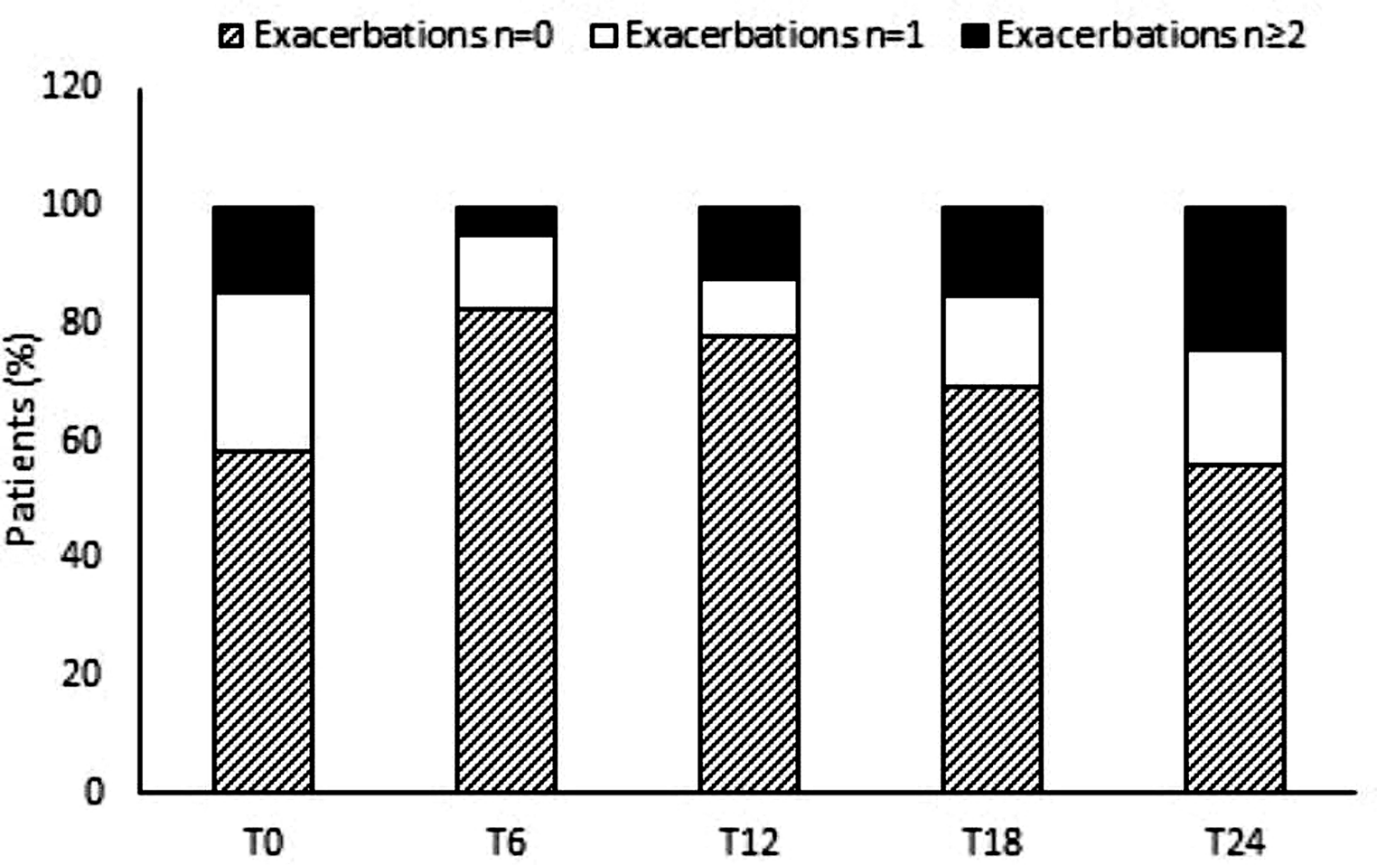
Percentages of subjects with or without exacerbations at the different time points (cumulative from T6). T0 shows the exacerbations reported by the patients in the year before enrolment

Cumulative exacerbations were 20 in 53 patients after 1 year, and 39 in 41 patients after 2 years, with significant difference at the different time-points considered (*p* < 0.0001), Fig. 3. Patients with at least 1 exacerbation at T24 did not differ from patients without exacerbations as concerns blood (% and count) and sputum eosinophils, Table 5.

**Table 5:** Blood (% and count) and sputum eosinophils after 2-year follow-up in subjects with and without exacerbations

T24	No exacerbation	≥ 1 exacerbation	*p*-value
Blood eosinophils, %	2.3 (1.4–3.5)	2.2 (1.8–3.5)	0.71
Blood eosinophils count	151.9 (90.1-233.3)	153.3 (120-217.2)	0.77
Sputum eosinophils, %	1.0 (0.7–1.9)	2.3 (0.9–3.2)	0.39

Legend: Data are expressed as median (IQR: interquartile range)

Among patients with 2-year follow-up, only 6/41 (14.6%) had blood eosinophil count ≥ 300/mcL, 1 experienced 2 exacerbations during the 2 years while 5 did not report exacerbations. At T0 14/53 patients had blood eosinophils ≥ 100 and exacerbations ≥ 1 and at T24 14/41, only 9 remained in bronchodilator therapy. None of the variables considered at T0 predicted exacerbations during the follow-up, neither did the history of exacerbations in the year before the enrollment (*p* = 0.09, OR: 2.08 95% CI 0.89–4.88).

### Comorbidities

Comorbidities of the enrolled patients are reported in Table 1. Patients with history of ischemic heart disease increased blood eosinophils at T24 compared to those without this comorbidity [delta blood eosinophils %: 10.5 (0.3–1.9) vs. -1 (-1; 0.5) *p* = 0.03 and blood eosinophils count: 72.2 (-8.1;169.7) vs. -12.9 (-46.5; 27.1) *p* = 0.03]. No other differences were found between patients with and without other comorbidities.

## Discussion

Changes in blood and sputum eosinophils in a prospective cohort of mainly mild to moderate COPD subjects were evaluated during a follow-up of 2 years. Patients were currently treated with single or dual bronchodilators and ICS was only added according to symptoms and exacerbations during the 2-year follow-up. ICS was added in a small proportion of subjects. Blood and sputum eosinophils mildly correlated at the recruitment and did not significantly change during the follow-up. There was no difference in delta (T24-T0) FEV_1_, cumulative exacerbations, delta blood and sputum eosinophils between patients with or without ICS treatment. Patients with history of ischemic heart disease showed increased blood eosinophils from T0 to T24.

Blood eosinophils is considered a treatable trait, since a better response to ICS treatment was found in subjects with blood eosinophilia [15–17]. In our population of mainly mild to moderate COPD, high blood eosinophils (both percentage and count) were not associated with an increased number of exacerbations. Only 14% of the subjects had blood eosinophils ≥ 300 cell/mcL at baseline and most of them did not report exacerbations during the follow-up. However, total exacerbations, slightly increased during follow-up but none of the variables considered at baseline predicted exacerbations. Jun JH et al. reported no associations between high blood eosinophil count and exacerbations in subjects with ≤ 1 exacerbation in the year before their study [15]. Kerkhof et al., found an increased risk of exacerbations only when blood eosinophils were ≥ 450 cells/mcL in ex-smoker COPD [18]. Casanova C et al. found that COPD patients with persistently high blood eosinophils did not experience increased exacerbations in a 2-year follow-up, and the pattern of blood eosinophil distribution was similar in COPD and in smoking subjects without COPD [19]. However, in our study, an indirect relationship between blood eosinophils and respiratory symptoms can be extrapolated from the increased blood eosinophil in subjects who received ICS treatment for occurrence of respiratory symptoms during follow-up. We did not find any correlations between blood eosinophils and variation of lung function during the follow-up, as in the ECLIPSE study [20].

Almost one third of our subjects (28.3%) showed high sputum eosinophils at baseline without any changes during the follow-up. A trend towards higher sputum eosinophils in subjects with ≥ 1 exacerbations after 2 year of follow-up was present, suggesting sputum eosinophils as a better biomarker in mild-moderate COPD than blood eosinophils. However, more data are needed to confirm this trend. Stability of low (< 1%) and intermediate (1–3%) sputum eosinophils was recently demonstrated in a 6-month monitoring study of COPD patients [21]. We found a poor correlation between blood and sputum eosinophils at baseline, in agreement with our previous study [2]. If blood eosinophils only reflect airway eosinophils in these patients, the poor correlation could be due to the localization of eosinophils in the small airways of COPD patients instead of in the central airways which are sampled by induced sputum technique [22].

In the SPIROMICS cohort, sputum eosinophils were found to be a better biomarker than blood eosinophils in identifying COPD patients with more severe disease, more frequent exacerbations, and increased emphysema; [8] however, the cut-off to define high sputum eosinophils is 1.25%, in the range of normal subjects [23]. We adopted a cut-off of 3% for sputum eosinophils, according to the median value of our population (1.7%, CQI: 0.7–3.4). Bartoli et al. in a retrospective study, found that COPD with sputum eosinophilia (≥ 2%) had a lower dyspnea score, a lower functional impairment, and a lower ICS use [24]. Sputum eosinophils were comparable at the different time points and ICS use did not affect blood or sputum eosinophils in our cohort of COPD patients as reported in other studies [25], however in contrast with some other studies [26–28]. As the association between increased blood eosinophils and exacerbation rate and/or response to ICS therapy could depend on the type of population analyzed, blood eosinophils cannot be a “generalized” biomarker [29].

A correlation between deterioration of lung function and sputum eosinophils was shown in a cohort of patients with mild chronic bronchitis [30]. We found that baseline FEV_1_% and FEV_1_/FVC were associated with ICS addition after the first and the second year of follow-up, respectively, suggesting that in mild/moderate COPD patients, lung function, rather than systemic or airway eosinophils, could be the driver of symptoms. Our data agreed with those of Hartjes FJ et al., who showed that baseline blood, sputum, bronchoalveolar lavage, and bronchial eosinophils as well as neutrophils were not associated with lung function deterioration in a population of ICS untreated COPD patients [9]. However, Kerkhof M et al. showed that when ICS untreated COPD patients with blood eosinophils ≥ 350 cell/mcL experienced an exacerbation, the subsequent FEV_1_ loss was higher than that experienced by patients with blood eosinophils ≥ 350/mcL and treated with ICS [31]. When we analyzed patients according to blood eos/mcL ≥ 100 and blood eosinophils/mcL 100–300, no differences in delta sputum eosinophils, delta FEV_1_ and exacerbations were found. These heterogeneous published data related to increased eosinophils and response to ICS should be considered to properly select COPD patients who really need ICS addition, considering the possible increase of severe pneumonia also reported with triple therapy [32].

Whether or not current smoking is associated with higher blood eosinophils is still controversial. Higher blood eosinophils were found in current smokers in the general population regardless of COPD presence [33, 34]. Higher eosinophils were instead found in smokers COPD compared with ex-smokers when BAL was evaluated [34]. Sputum, BAL and bronchial tissue eosinophils did not change even after smoking cessation at least in a small population of COPD subjects evaluated 1 year after quitting smoking [35]. In our study, smoking history does not seem to have a significant impact on symptoms and prediction of ICS addition, as previously reported [36].

Patients with a history of ischemic heart disease had increased blood eosinophils during the follow-up, confirming our previous data in another group of COPD patients [2]. Increased blood eosinophils were reported as risk factor for coronary heart disease [37], and a direct correlation between coronary heart calcification and increased blood eosinophils was proved by Tanaka M et al. [38]. Furthermore, patients without COPD but with acute ischemic stroke had higher blood eosinophils [39]. Moreover, in our study 10/63 patients dropped out mainly for acute cardiovascular events and these subjects had higher blood eosinophils than those who were included in the study.

Our study has some limitations: the sample size is modest and the study is monocentric. However, to our knowledge, this is the first prospective study monitoring every 6 months, for two years, sputum other than blood eosinophils in mild-moderate COPD patients. Furthermore, we excluded patients with a previous asthma diagnosis and most of the enrolled subjects were routinely treated with bronchodilators and not ICS-washed out.

## Conclusion

Mild-moderate COPD patients only treated with bronchodilators maintain stable blood and sputum eosinophils after 1 or 2-year follow-up. A small proportion of them needed ICS addition due to increased symptoms.

## Data Availability

The datasets used and/or analysed during the current study available from the corresponding author on reasonable request.

## Abbreviations

CAT: COPD Assessment Test
CI: Confidence Interval
COPD: Chronic Obstructive Pulmonary Disease
FEV_1_: Forced Expiratory Volume in the First Second
FVC: Forced Vital Capacity
GOLD: Global Initiative for Chronic Obstructive Lung Disease
ICS: Inhaled Corticosteroids
IQR: Interquartile Range
LAMA: Long-Acting Muscarinic Antagonists
LABA: Long-Acting β2-Agonist
OR: Odds Ratio
OSAS: Obstructive Sleep Apnea Syndrome
